# Epigenetic Pathways Promoting Immune Evasion in Human Papillomavirus-Positive Head and Neck Squamous Cell Carcinoma

**DOI:** 10.51894/001c.143851

**Published:** 2025-10-01

**Authors:** Nicholas S. Giacobbi, Lexi Vu, Canchai Yang, Shreya Mullapudi, Will Eckerman, John Vusich, Eran Andrechek, Mohamed Khalil, Andrew Olive, Dohun Pyeon

**Affiliations:** 1 College of Osteopathic Medicine Michigan State University; 2 College of Osteopathic Medicine Michigan State University

## 10

### INTRODUCTION

Human papillomavirus-positive head and neck squamous cell carcinoma (HPV+ HNSCC) exhibits reduced MHC-I expression for antigen presentation, leading to immune evasion and limited efficacy of immune checkpoint inhibitor (ICI) therapy.

### OBJECTIVES

In HPV+ HNSCC, we sought to identify negative regulators of MHC-I expression and to elucidate the underlying mechanism(s) of downregulation. Identifying new targets to increase MHC-I antigen presentation should improve ICI therapy efficacy.

### METHODS

We performed genome-wide CRISPR/Cas9 screens in HPV+ HNSCC cell lines and identified members of the polycomb repressive complex 2 (PRC2) as significant negative regulators of MHC-I. PRC2 is known to repress MHC-I-related gene expression via histone methylation (H3K27me3) in other cancers, and expression of the catalytic core protein of PRC2, EZH2, is upregulated in HPV+ cancers. We hypothesized that PRC2-mediated methylation leads to transcriptional downregulation of MHC-I expression in HPV+ HNSCC.

### RESULTS

We found increased expression of PRC2 and H3K27me3 levels in HPV+ HNSCC cells. Interestingly, our pathway analysis identified the Spt-Ada-Gcn5 acetyltransferase (SAGA) complex as the top gene network responsible for the downregulation of MHC-I expression. The SAGA complex, via the histone lysine acetyltransferase (KAT2A), is a transcriptional coactivator for E2F and MYC targeted transcription, whose activity is highly upregulated in HPV+ HNSCC cells via the viral oncoproteins E7 and E6. We show that KAT2A and SAGA complex expression is upregulated in HPV+ HNSCC, and that the inhibition of PRC2, KAT2A, or other SAGA complex members resulted in increased MHC-I expression. By extension, our analysis identified several compounds in various stages of clinical development which could be used to augment the current standard of care for HPV+ HNSCC cells.

### CONCLUSIONS

Our results suggest that elevated KAT2A/SAGA potentiates E2F- and MYC-mediated transcription of PRC2 genes, contributing to the downregulation of MHC-I. Thus, abrogation of the KAT2A/SAGA:E2F/MYC:PRC2 axis may be valuable toward increasing antitumor immunity through the restoration of MHC-I expression in HPV+ HNSCC.

